# Déhiscence de la lame papyracée

**DOI:** 10.11604/pamj.2019.34.77.18675

**Published:** 2019-10-10

**Authors:** Sameh Mezri, Sameh Sayhi

**Affiliations:** 1 Service d’Oto-Rhino-Laryngologie, Hôpital Militaire de Tunis, Tunis, Tunisie; 2 Service de Médecine Interne, Hôpital Militaire de Tunis, Tunis, Tunisie

**Keywords:** Lamina papyracea, dehiscence, emphysema, Lame papyracée, déhiscence, emphysème

## Abstract

We report the case of a 35 year old patient presenting with acute onset of right periocular pain with palpebral edema due to nose blowing with absence of trauma. Early assessment showed periorbital emphysema without ophthalmological involvement, normal pupillary light reflex and visual acuity. Computerized tomography (CT) scan of facial and orbital bones objectified right pneumo-orbita with orbital fat herniation via a partial agenesis of the lamina papyracea, without muscle incarceration. Emphysema gradually reduced after two weeks by applying pressure dressing associated with broad spectrum antibiotic therapy as well as following the rules for cleanliness (avoiding closed glottis efforts, nose blowing…). Pneumo-orbita is defined by the presence of orbital air. It is mainly caused by a trauma but few cases of spontaneous dehiscence have been reported. The opening of the lamina papyracea, acting as an antireflux valve, prevents air outlet. CT scan is essential to confirm the diagnosis. It most often has a spontaneous resolution but the presence of hyperpression, that can be responsible for ischemic optic neuropathy or occlusion of the central artery of the retina, require a rigorous monitoring in order to implement possible urgent decompressive intervention.

## Image en médecine

Nous rapportons le cas d'un patient âgé de 35 ans ayant consulté pour installation brutale d'une douleur périoculaire droite avec un œdème palpébral faisant suite à un effort de mouchage avec absence d'un contexte traumatique. L'examen initial a objectivé un emphysème périorbitaire sans retentissement ophtalmologique, un reflexe photomoteur présent et une acuité visuelle normale. Le scanner du massif facial et orbitaire a objectivé une pneumorbitie droite avec une hernie de la graisse orbitaire à travers une agénésie partielle de la lame papyracée sans incarcération musculaire. L'emphysème s'est progressivement résorbé au bout de deux semaines en appliquant un pansement compressif avec mise sous antibiothérapie à large spectre et en respectant les règles d'hygiène (éviter les efforts à glotte fermée, le mouchage,…). La pneumorbitie est définie par la présence d'air dans le cadre orbitaire. L'origine est généralement traumatique mais quelques cas de déhiscence spontanée ont été rapportés. L'ouverture de la lame papyracée, agissant comme une valve anti reflux, empêche la sortie d'air. Le scanner est primordial pour confirmer le diagnostic. L'évolution est le plus souvent spontanément résolutive mais la présence d'une hyperpression pouvant être responsable d'une neuropathie optique ischémique ou une occlusion de l'artère centrale de la rétine rendent nécessaire une surveillance rigoureuse pour une éventuelle intervention décompressive urgente.

**Figure 1:**
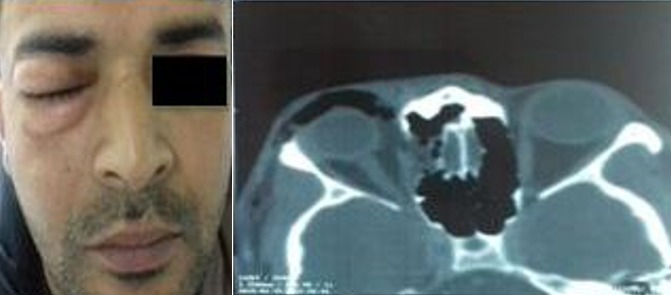
Emphysème palpébral droit, avec à la TDM orbitaire (coupe axiale) une pneumorbitie droite et une hernie de la graisse orbitaire à travers une agénésie partielle de la lame papyracée

